# Yoga exercises can improve sustained attention in children with ADD

**DOI:** 10.1192/j.eurpsy.2021.569

**Published:** 2021-08-13

**Authors:** S. Kiselev

**Affiliations:** Clinical Psychology, Ural Federal University, Ekaterinburg, Russian Federation

**Keywords:** sustained attention, attention deficit disorder, yoga exercises

## Abstract

**Introduction:**

It is known that children with attention deficit disorder (ADD) have deficit in sustained attention. It is important to develop trainings for improving sustained attention in ADD children. It was shown that yoga exercises have positive effect on sustained attention in adults.

**Objectives:**

The gaol of this study was to reveal effect of yoga exercises on sustained attention in 7-8 years of age children with ADD. We compared the efficacy of two methods of treatment (yoga exercises vs. conventional motor exercises) in a randomized controlled pilot study.

**Methods:**

18 children with ADD at the age of 7-8 years were included and randomly assigned to treatment conditions according to a 2×2 crossover design. Children from intervention group participated in 8 weeks of yoga exercises. To assess the sustained attention we used subtest from Luria’s child neuropsychological battery. This subtest is designed to assess visual sustained attention.. Effects of treatment were analyzed by means of an ANOVA for repeated measurements.

**Results:**

The ANOVA has revealed (p<.05) that for sustained attention subtest the yoga exercises were superior to the conventional motor training, with effect sizes in the medium-to-high range (0.39-0.77).

**Conclusions:**

The findings from this pilot study suggest that yoga exercises have positive effect on sustained attention in 7-8 years of age children with ADD. However, it is necessary to do further research to reveal the impact of yoga exercises on the prevention and treatment of attention deficit disorder in children.

